# Rural Versus Urban Health Service Utilization and Outcomes for Renal Patients in New South Wales: Protocol for a Data Linkage Study

**DOI:** 10.2196/resprot.3299

**Published:** 2015-06-16

**Authors:** Sradha Kotwal, Angela Webster, Alan Cass, Martin Gallagher

**Affiliations:** ^1^ The George Institute for Global Health Sydney Medical School University of Sydney Sydney Australia; ^2^ Sydney School of Public Health University of Sydney Sydney Australia; ^3^ Centre for Transplant and Renal Research Westmead Hospital Sydney Australia; ^4^ Menzies School of Health Research Charles Darwin University Darwin Australia; ^5^ Concord Clinical School Sydney Medical School The University of Sydney Sydney Australia

**Keywords:** end stage kidney disease, chronic kidney disease, kidney transplant, data linkage, dialysis, rural health care, cohort study

## Abstract

**Background:**

Kidney disease is a significant burden on health systems globally, with the rising prevalence of end stage kidney disease in Australia mirrored in many other countries. Approximately 25% of the Australian population lives in regional and rural areas and accessing complex tertiary services is challenging.

**Objective:**

We aim to compare the burden and outcomes of chronic kidney disease and end stage kidney disease in rural and urban regions of New South Wales (Australia’s most populous state) using linked health data.

**Methods:**

This is a retrospective cohort study and we have defined two cohorts: one with end stage kidney disease and one with chronic kidney disease. The end stage kidney disease cohort was defined using the Australia and New Zealand Dialysis and Transplant Registry, identifying all patients living in NSW receiving renal replacement therapy at any time between 01/07/2000 and 31/07/2010. The chronic kidney disease cohort used the NSW Admitted Patient Data Collection (APDC) to identify patients with a diagnostic code relating to chronic renal failure during any admission between 01/07/2000 and 31/07/2010. Both cohorts were linked to the NSW APDC, the Registry of Births, Deaths and Marriages, and the Central Cancer Registry allowing derivation of outcomes by categories of geographical remoteness.

**Results:**

To date, we have identified 10,505 patients with 2,384,218 records in the end stage kidney disease cohort and 159,033 patients with 1,599,770 records in the chronic kidney disease cohort.

**Conclusions:**

This study will define the geographical distribution of end stage and chronic kidney disease and compare the health service utilization between rural and urban renal populations.

## Introduction

Kidney disease is a significant burden upon health systems globally. The rate of new end stage kidney disease (ESKD) cases in 2012 was 357 per million in the United States, 108 in the United Kingdom and 110 in Australia [1-3]. The overall incidence of treated ESKD in Australia has increased by 19%, between 2000 and 2007 [4]. This increasing burden of disease is largely borne by older Australians, with prevalence rates much higher in those aged 65-84 years [3]. As the population ages, it is likely to drive further increases in the prevalence of ESKD.

The cost of renal service provision in the United States in 2010 was US$47.5 billion [1] and is expected to rise to $1.5 to 1.8 billion by 2019 in Australia [5]. Dialysis is the most common reason for hospitalization in Australia, and chronic kidney disease (CKD) contributed to 15% (1.2 million) of hospitalizations in Australia in 2007 and 2008. [5].

New South Wales (NSW) is Australia’s most populous state and includes 32.3% of Australia’s population, with approximately 25% living in rural and remote areas. There is currently inadequate data regarding differences in growth in demand for renal replacement therapy (RRT) in rural versus urban areas in Australia [6]. Although it has been documented that increasing remoteness corresponds to increasing incidence of ESKD amongst indigenous Australians, such geographic patterns have not been well defined for non Indigenous Australians [7]. This is despite the fact that nationally non Indigenous Australians constitute the majority of ESKD patients in all regions except remote areas. A United States Renal Data Service (USRDS) analysis published in 2006 found a geographic difference in access to types of RRT, with rural facilities less likely to offer home based therapies, but did not explore many other important elements of nephrology service access (eg, dialysis access creation, distance to nephrology services) [8]. Poorer outcomes for patients with increasing distance from nephrology services [9,10] have been documented internationally, but this has not been examined in an Australian context. American, Canadian and Australian studies show that there is a reduced access to kidney transplantation in remote and rural areas, but differences in access to other forms of renal replacement therapy are poorly delineated [11-15].

The Australian Diabetes, Obesity and Lifestyle Survey (AUSDiab) estimated that approximately 16% of the Australian adult population has a marker indicating the presence of kidney damage [16]. This study was conducted in a community-based cohort. There is limited information regarding the health service use and burden of disease of those with CKD especially for those that live in rural and remote areas. A further challenge in nephrology care is that 21% of all patients in Australia starting ESKD treatment programs are referred ‘late’ to nephrological care (ie less than 3 months before first RRT [3]. There is currently a paucity of data on the geographical distribution of late referral and given that the majority of tertiary nephrology services are provided in large urban areas, areas with fewer nephrologists would appear especially vulnerable to this problem.

Validation of administrative datasets with renal disease specific registries has been conducted in Australia and a high level of agreement between the two collections was found [17-20]. This suggests that the use of an administrative dataset combined with a disease specific registry will allow us to estimate the geographical distribution of kidney disease and derive data on outcomes and burden of both CKD and ESKD. This study will use data linkage of clinical and administrative datasets to study the difference in health service utilization and outcomes between rural and urban CKD and ESKD patients in NSW.

## Methods

### Overview

Our study hypotheses are that rural patients with ESKD and CKD have higher mortality, higher hospitalization rates, and longer lengths of stay, require more inter-hospital transfers and have higher rates of late referral for RRT compared to similar urban patients. We expect that in an Australian setting, rural patients with ESKD use home-based therapies more often than urban patients, despite evidence to the contrary in a North American setting. We also expect that rural patients with CKD or ESKD and at least one other comorbid condition (cardiovascular disease, diabetes or cancer) have a greater burden of disease defined as a higher mortality, higher hospitalization rates, longer lengths of stay and more requirements for inter-hospital transfer compared to similar urban CKD and ESKD patients.

### Study Population

**Figure 1:**
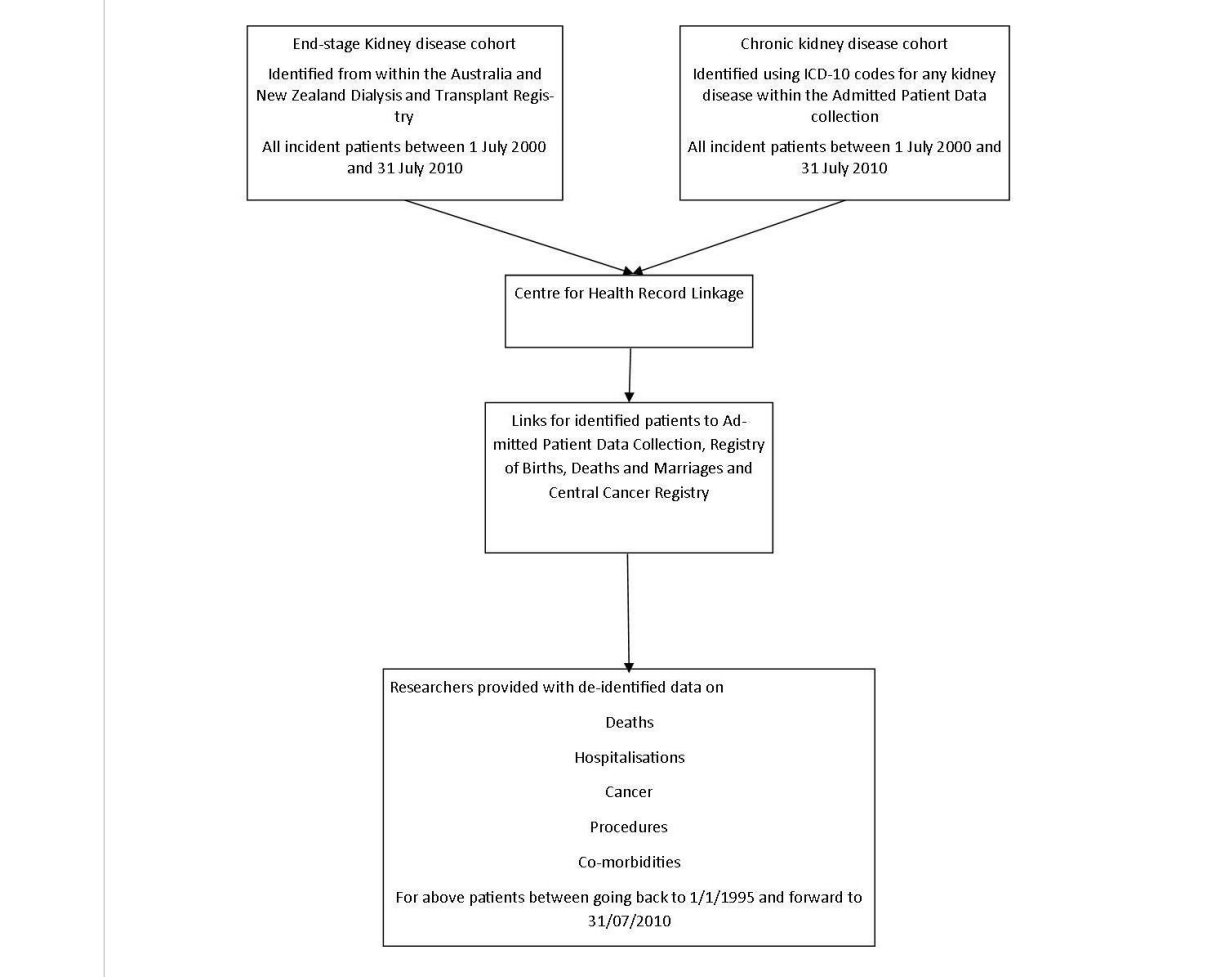
Data linkage process chart.

This is a retrospective cohort study consisting of two cohorts (see Figure 1); the first an ESKD cohort and the second a CKD cohort.

The ESKD cohort will be identified using the Australia and New Zealand Dialysis and Transplant Registry (ANZDATA). This registry was established in 1963 and maintains records of all patients with ESKD receiving chronic renal replacement therapy (dialysis or transplantation) in Australia and New Zealand. All patients residing in NSW at initiation of ESKD treatment between 1/7/2000 and 31/07/2010 will be included in the ESKD cohort.

The CKD cohort will be identified from within NSW Admitted Patient Data Collection (NSW APDC) by the Centre for Health Record Linkage (CHeReL) [21], and will be defined as any patient admitted to a NSW hospital between 1/07/2000 to 31/07/2010, with a recorded admission using International Classification of Diseases 10 - Australian Modification (ICD-10AM) primary or secondary codes for chronic renal failure or chronic renal impairment including transplantation (Table 1). Patients with ESKD that are in receipt of RRT will also be identified within the CKD cohort. If these patients are also part of the ESKD cohort, in other words identified via ANZDATA, then they will be tagged during record linkage as belonging to the ESKD cohort. Those patients with ESKD that are not in receipt of renal replacement therapy will only be identified as part of the CKD cohort because ANZDATA only records patients that are in receipt of renal replacement therapy.

Both cohorts will be linked to NSW Admitted Patients Data Collection (NSW APDC), the NSW Registry of Births, Deaths and Marriages (NSW RBDM), and the NSW Central Cancer Registry (NSW CCR). The NSW APDC records all admissions to all NSW health care facilities, the NSW RBDM records all births, deaths and marriages within NSW and the NSW CCR records all new cancers in NSW residents.

Those that are under the age of 18 at the commencement of RRT or at the time of their first admission with a code for CKD will be excluded as well as those that do not normally reside in NSW. Residence will be assessed on the basis of postal code at the commencement of RRT or at the first admission with a code for CKD.

**Table 1:** ICD-10AM^a^ codes used to identify CKD cohort.

ICD 10 code	Description
N18.1	Chronic kidney disease stage 1
N18.2	Chronic kidney disease stage 2
N18.3	Chronic kidney disease stage 3
N18.4	Chronic kidney disease stage 4
N18.5	Chronic kidney disease stage 5
N18.8	Other Chronic renal failure
N18.9, N18.90, N18.91	Chronic kidney disease unspecified
N19	Unspecified renal failure
N 16.0-N16.8	Renal tubulo-interstitial disorders in diseases classified elsewhere
I12.0, I13.1, I13.2	Hypertensive kidney disease with kidney failure
E10.2, E11.2, E12.2, E13.2, E14.2	Diabetes with kidney complication
N00-N07	Chronic nephritic syndrome, Nephrotic syndrome
N11.0, N11.1, N11.8, N11.9, N12	Chronic tubulo-interstitial nephritis
N14.0-N14.4	Drug and other tubular conditions such as analgesic nephropathy
N25.0, N25.1, N25.8, N25.9, N26	Impaired tubular function and unspecified contracted kidney
N27.0, N27.1, N27.9	Small contracted kidney
N28.0, N28.1, N28.8, N28.9	Other disorders of kidney not elsewhere specified
N39.1	Persistent proteinuria
N39.2	Orthostatic proteinuria
B52.0	Plasmodium with nephropathy
D59.3	Hemolytic uremic syndrome
E85.3	Secondary systemic amyloidosis
Q60.0-Q60.6	Renal agenesis
Q61.3	Polycystic kidney disease unspecified
T 82.4	Mechanical complication of vascular dialysis catheter
T86.1	Kidney transplant failure and rejection
Z94.0	Renal transplant

^a^International classification for diseases 10 – Australian Modification

### Exposures and Outcomes

The exposure is rural residence defined using the Accessibility/Remoteness Index of Australia (ARIA) [22]. ARIA provides a measure of remoteness (from service centers) for all places and points in Australia using Geographic Information System (GIS) technology and was developed by the Commonwealth Department of Ageing and Health Care. Categories of remoteness are defined based on road distance to service centers and are: highly accessible (relatively unrestricted accessibility to goods and services), accessible (some restrictions to accessibility of some goods and services), moderately accessible (significant restriction to accessibility to goods and services), remote (very restricted accessibility to goods and services) and very remote (very little accessibility of goods and services). We will use residential postal codes to classify people into categories of remoteness. It is expected that the majority of postcodes will be located within the highly accessible (urban) areas and thus we will define this group as the index group [23]. All other postcodes will be considered as rural. There is no data currently on the geographical distribution of the burden of disease and thus we may need to either combine or separate categories depending on their size.

For the ESKD and the CKD cohorts, the following outcomes will be compared amongst the categories of remoteness: mortality (derived from fact and date of death via the NSW RBDM); hospitalizations (number of hospitalizations and location of hospitalization derived from NSW APDC); length of stay (using the hospitalization data provided by NSW APDC); inter-hospital transfers (calculated using the admission and discharge data gained from the NSW APDC). For the ESKD cohort, an additional outcome of rate of late referral to specialist care (identified via ANZDATA using the late referral flag, which measures those referred to nephrology care who subsequently start RRT within 3 months) and patterns of use of RRT (identified and compared using data on modalities of RRT used by patients from within ANZDATA) will also be compared between the categories of remoteness.

For both cohorts (ESKD and CKD), we will identify those with an additional diagnosis of cardiovascular disease (ICD10-AM Codes: I00-I52.8, I170 to I99), diabetes (ICD10-AM Codes: E10-14), or cancer (ICD10-AM Codes: C00-D48) and compare the outcomes of mortality, hospitalizations, lengths of stay and inter-hospital transfers as defined above.

### Data Linkage Methods

Data linkage is probabilistic using demographic markers such as name, date of birth, gender, country of birth, medical record number (MRN), date of first RRT, postcode at first RRT, treating hospital and date of death to link patients identified by ANZDATA to the NSW APDC, NSW RBDM and the NSW CCR [24]. All admissions from NSW hospitals going forward to 31/07/2010 and backwards to 1/1/1995 for patients in the two cohorts will be identified using NSW APDC and any diagnosis of cancer will be identified using NSW CCR. Fact of death and date of death will be ascertained using NSW RBDM.

Data linkage will be performed using the services and processes of the CHeReL. CHeReL was established in 2006 with the aim of linking multiple sources of data and maintaining a record linkage system that protects data privacy and is jointly managed by the Cancer Institute NSW and the NSW Ministry of Health. Each data custodian provides information relating to individual persons to the CHeReL. This information consists solely of personally identifying information, plus an encrypted source record number (which is the link to the health dataset records). CHeReL uses the personally identifying information to link records for the same person across different datasets, and assigns a ‘person number’ to each of these groups of linked records (note that this ‘person number’ never leaves the CHeReL). CHeReL then develops a set of ‘project person numbers’ (PPN), which identifies all the records that correspond to a single person. CHeReL uses the *Choicemaker* software package to link records. Clerical review is also conducted for records with doubtful matches, resulting in a false positive rate of <0.5% [25].

Once the required linkage has been completed with the groups of linked records identified and PPNs allocated, the CHeReL removes all identifiable information from the linked data sets and sends the data back to the respective data custodians. The data sent to the custodians contains their own encrypted source record numbers plus corresponding PPNs. The PPNs indicate which records correspond to a single individual so that the researchers can combine data from the different data custodians. Each data custodian then removes the source record numbers, and provides the researchers with the PPNs and the associated requested health data. This process ensures that the researchers are provided with deidentified data in which re-identification is effectively impossible.

### Statistical Analysis

We will separate the ESKD and CKD cohorts into categories of remoteness using the ARIA index as explained above [22]. We expect approximately 10,000 patients in the ESKD cohort and approximately 100,000 patients in the CKD cohort, however the CKD cohort is difficult to estimate accurately as there is scant data available on the prevalence of CKD in an admitted patient cohort in Australia. We expect approximately 25% of both cohorts to live outside of urban areas. Baseline characteristics for patients within both cohorts will be compared using *t* test, chi-square and ANOVA. The association between remoteness and mortality will be explored deriving hazard ratios and 95% CIs using Cox proportional hazards models. Hospitalizations and inter-hospital transfers will be compared using logistic regression and Poisson regression. The length of stay outcome, being a continuous variable, will be analyzed using linear models. All models will be multivariable to adjust for demographic variables, comorbid conditions, and geographical access to services. We estimate that our study is powered to detect at least a 5% mortality difference between the urban and rural cohort with at least 90% power and a 0.05 level of significance with 10,000 ESKD and 100,000 CKD patients of which 75% are urban and 25% are rural. Stata 12.1 will be used for analysis and a two - tailed *P* value of <0.5 is set as the level of significance.

### Ethical Considerations

This study has been granted ethical approval in January 2012 by the NSW Population & Health Services Research Ethics Committee along with approval from all data custodians. As no identifiable data will be provided to the investigators the risk to privacy of participants from the misuse of personal information used in the record linkage process is extremely small. This risk is further minimized by separating the processes of record linkage and data analysis. All data will be reported in aggregated form and no reports or presentations will identify any individual or organization.

The linkage keys, which allow linking of the relevant datasets, are destroyed 12 months following the supply of the data. After this time there will be no potential to reidentify the data. The data will be stored on secure servers for five years to enable the researchers to answer any queries arising from the publications as per ethical approval.

## Results

### Overall Population

11,036 patients were identified by ANZDATA, of whom 10,827 patients also had records within NSW APDC. A further 322 patients either had missing postcodes or a non-NSW postcode leaving a total of 10,505 patients with 2,403,455 records in the ESKD cohort. Based on the ARIA categories, 85.46% of patients (8978/10,505) live in highly accessible areas; 11.77% (1236/10,505) in accessible areas; 1.84% (193/10,505) in moderately accessible areas; 0.66% (69/10,505) in remote areas and 0.28% (29/10,505) in very remote areas. For the purposes of analysis, patients living in accessible, moderately accessible, remote and very remote areas were combined as the rural cohort – 14.54% (1527/10,505).

The CKD cohort comprised of 164,236 patients. Exclusion of patients with missing or non-NSW postcodes resulted in 159,033 patients with 1,599,770,776 records in this cohort. Based on ARIA categories, 84.05% (133,667/159,033) live in highly accessible areas; 13.14% (20,904/159,033) in accessible areas; 2.05% (3260/159,033) in moderately accessible areas; 0.65% (1027/159,033) in remote areas and 0.11% (175/159,033) in very remote areas. For the purposes of analysis, patients living in accessible, moderately accessible, remote and very remote areas were combined as the rural cohort – 15.95% (25,366/159,033). The baseline characteristics of both cohorts are detailed in Table 2.

**Table 2:** Baseline characteristics of ESKD and CKD patients in New South Wales between 01/07/2000 and 31/07/2010.

	ESKD (Urban) n=8978 (85%)	ESKD (Rural) n=1527 (15%)	*P* value for difference	CKD (Urban) n=133,667 (84.1%)	CKD (Rural) n=25,366 (15.95%)	*P* value for difference
Age (median & IQR)	61 (48-72)	61 (48-71)	.43	75.0 (62-83)	74.0 (62-81.8)	<.001
Male (%)	5246 (58.43%)	888 (58.15%)	.84	69,142 (51.73%)	13,392 (52.80%)	.002
Indigenous Australians (%)	157 (1.75%)	166 (10.87%)	<.001	1161 (0.87%)^a^	1143 (4.55%)^a^	<.001
Comorbidities (%)						
Diabetes (From ANZDATA)	2745 (30.57%)^b^	454 (29.73%) ^b^	.51	NA	NA	NA
(From NSW APDC)	1536 (17.11%) ^c^	310 (20.30%)^c^	.002	43,072 (32.22%)	8,019 (31.61%)	.06
Cardiovascular disease (From ANZDATA)	3164 (35.24%)^b^	592 (38.77%) ^b^	.008	NA	NA	NA
(From NSW APDC)	1237 (13.78%)^c^	220 (14.41%)^c^	.51	46,137 (34.52%)	8,355 (32.94%)	<.001
Peripheral vascular disease (From ANZDATA)	2060 (22.94%)^b^	440 (28.81%) ^b^	<.001	NA	NA	NA
(From NSW APDC)	3 (0%)^c^	1 (0%)^c^	.55	107 (0.08%)	9 (0%)	.02
Chronic lung disease (From ANZDATA)	1273 (14%) ^b^	291 (19%) ^b^	<.001	NA	NA	NA
(From NSW APDC)	198 (2.2%)^c^	41 (3%)^c^	.25	11,545 (8.6%)	2,256 (8.9%)	.18

^a^Recorded for 157,792 (99.21%) patients.

^b^For the ESKD cohort, these were derived from ANZDATA.

^c^Derived using ICD – 10 codes from the NSW Admitted Patient Data Collection. For the CKD cohort these were derived using ICD-10 codes for the index admission and in any admission prior to the index admission from within the NSW Admitted Patient Data Collection. The ICD – 10 codes were as per AIHW: *Australian Institute of Health and Welfare 2011. Cardiovascular disease: Australian facts 2011. Cardiovascular disease series. Cat. no. CVD 53. Canberra: AIHW.*

### Results of Data Linkage

The mortality linkage identified a total of 96,313 records (88,020 patients) comprising 5463 records (5028 patients) in the ESKD cohort and 90,850 records (82,992 patients) in the CKD cohort. The linkage with the NSW CCR identified a total of 40,668 cancer records (36,110 patients) comprised of 1905 records (1693 patients) in the ESKD cohort and 38,763 records (34,417 patients) in the CKD cohort.

## Discussion

### Anticipated Outcomes

This research which has identified 11,036 ESKD patients and 164,236 patients in the CKD cohort will define the geographical distribution of CKD and ESKD as well as the demand for RRT in the NSW population. It will compare and contrast health service utilization between rural and urban populations with a view to informing the design and implementation of strategies to provide appropriate rural health care in the future. We will be able to delineate areas of higher incidence and prevalence and aid prediction of the need for future renal services. Given that 32.3% of the Australian population resides in NSW, this research has relevance for renal policy nationally.

The ANZDATA registry has made significant contributions to our understanding of kidney disease. This study expands the scope of ANZDATA and therefore will increase our insight into the drivers of mortality and poor outcomes in the kidney disease population. The ANZDATA registry however only records patients with ESKD that commence RRT and there has been no avenue previously for obtaining data on those with CKD/ESKD who are not receiving RRT except in the context of clinical trials. Our study allows us to comment on longitudinal outcomes in treated and untreated ESKD patients in a geographical context.

A notable limitation of our study however, is that the ascertainment of CKD relies purely on coding practices and coding intensity. Whilst there is no Australian data estimating prevalence of CKD in an admitted patient cohort, making it difficult to comment on the accuracy of coding for the CKD cohort, this dataset will be an important baseline for future research. Linkage with the ANZDATA registry for the ESKD cohort provides us the opportunity to report on validation of coding for the ESKD cohort as well as their comorbidities. Administrative health data, such as that used in this study, may represent a cheaper and effective alternative to performing large de novo longitudinal studies or maintaining large datasets. If so, it may also be a sustainable long-term option for measurement of disease burden and informing service delivery. A further strength is that because Australia has universal health coverage, our study includes all patients with kidney disease over a 10 year period that have had contact with private or public health care facilities in NSW.

### Conclusions

This is a large retrospective Australian cohort study of patients with ESKD and CKD that uses the linkage of an existing renal registry and administrative datasets to compare the burden and outcomes of kidney disease in rural compared to urban settings. The results will enhance our understanding of the capability of administrative data in measuring kidney disease in Australia, compare the burden and outcomes in patients with kidney disease between rural and urban settings, and contribute to the design and development of renal health service provision in future years.

## Abbreviations

ANZDATA: Australia and New Zealand Dialysis and Transplant Registry
ARIA: Accessibility/Remoteness Index of Australia
AUSDiab: Australian Diabetes, Obesity and Lifestyle Survey
CHeReL: Centre for Health Record Linkage
CKD: chronic kidney disease
DOB: date of birth
ESKD: end stage kidney disease
GIS: Geographic Information System
HREC: Human Resources Ethics Committee
ICD-10AM: International Classification of Diseases 10 – Australian Modification
MRN: Medical Record Number
NSW: New South Wales
NSW APDC: New South Wales Admitted Patient Data Collection
NSW CCR: New South Wales Central Cancer Registry
NSW RBDM: New South Wales Registry of Births Marriages and Deaths
PPN: project person numbers
RRT: Renal Replacement Therapy
USRDS: United States Renal Data Service
